# Paracellular Absorption: A Bat Breaks the Mammal Paradigm

**DOI:** 10.1371/journal.pone.0001425

**Published:** 2008-01-09

**Authors:** Enrique Caviedes-Vidal, William H. Karasov, Juan Gabriel Chediack, Verónica Fasulo, Ariovaldo P. Cruz-Neto, Lye Otani

**Affiliations:** 1 Laboratorio de Biología “Prof. E. Caviedes Codelia”, Facultad de Ciencias Humanas, Universidad Nacional de San Luis–Consejo Nacional de Investigaciones Científicas y Técnicas (CONICET), San Luis, Argentina; 2 Departamento de Bioquímica y Ciencias Biológicas, Universidad Nacional de San Luis, San Luis, Argentina; 3 Department of Wildlife Ecology, University of Wisconsin - Madison, Madison, Wisconsin, United States of America; 4 Área de Psicobiología, Facultad de Ciencias Humanas, Universidad Nacional de San Luis, San Luis, Argentina; 5 Departamento de Zoologia, Instituto de Biociências, Universidade Estadual Paulista, Rio Claro, São Paulo, Brazil; University of Sheffield, United Kingdom

## Abstract

Bats tend to have less intestinal tissue than comparably sized nonflying mammals. The corresponding reduction in intestinal volume and hence mass of digesta carried is advantageous because the costs of flight increase with load carried and because take-off and maneuverability are diminished at heavier masses. Water soluble compounds, such as glucose and amino acids, are absorbed in the small intestine mainly via two pathways, the transporter-mediated transcellular and the passive, paracellular pathways. Using the microchiropteran bat *Artibeus literatus* (mean mass 80.6±3.7 g), we tested the predictions that absorption of water-soluble compounds that are not actively transported would be extensive as a compensatory mechanism for relatively less intestinal tissue, and would decline with increasing molecular mass in accord with sieve-like paracellular absorption. Using a standard pharmacokinetic technique, we fed, or injected intraperitonealy the metabolically inert carbohydrates L-rhamnose (molecular mass = 164 Da) and cellobiose (molecular mass = 342 Da) which are absorbed only by paracellular transport, and 3-O-methyl-D-glucose (3OMD-glucose) which is absorbed via both mediated (active) and paracellular transport. As predicted, the bioavailability of paracellular probes declined with increasing molecular mass (rhamnose, 90±11%; cellobiose, 10±3%, n = 8) and was significantly higher in bats than has been reported for laboratory rats and other mammals. In addition, absorption of 3OMD-glucose was high (96±11%). We estimated that the bats rely on passive, paracellular absorption for more than 70% of their total glucose absorption, much more than in non-flying mammals. Although possibly compensating for less intestinal tissue, a high intestinal permeability that permits passive absorption might be less selective than a carrier-mediated system for nutrient absorption and might permit toxins to be absorbed from plant and animal material in the intestinal lumen.

## Introduction

Among mammals, bats are unique in their capacity for flapping flight, which requires high rates of energy expenditure. Small bats (10–50 g) eat as much or more than most of their similar-sized nonflying relatives [1]. Bats do have respiratory refinements that adapt them for flight [2], and it might be expected that they have digestive refinements for extracting energy from food at high rates. But, the demands of flight may shape an aspect of bats' digestive apparatus in a way that runs counter to that system's role in providing fuel to meet high energy demand. Biologists familiar with comparative digestive anatomy have noted that when contrasting comparably sized bats and non flying mammals without taken into account phylogeny, tend to have less intestinal tissue than nonflying mammals [3]–[5]. This pattern was confirmed in our allometric comparisons of gastrointestinal tract morphometrics of bats and nonflying mammals (E.C-V and W.H.K, unpublished data). Minimizing size of the digestive organ and consequently the digesta load it carries might be advantageous because the costs of flight increase with load carried [6] or because take-off and maneuverability may be diminished or even impaired at heavier masses [7], [8]. A central dilemma that emerges in this consideration of bats vs. nonflying mammals is how bats satisfy relatively high energy needs with relatively low intestinal nominal surface area for absorption.

Water soluble compounds, such as glucose and amino acids, are absorbed in the small intestine mainly via two pathways, the transcellular and the paracellular pathways. The first has been demonstrated to account for most of intestinal water-soluble nutrient absorption in several non flying mammal species [9]–[11] while the second has been found to be substantial only in birds [12]–[16]. The transcellular absorption of sugars and amino acids is primarily mediated by membrane-bound transporter proteins that take them up from the gut lumen into the enterocyte across the apical membrane, and hasten their exit from enterocyte to blood across the basolateral membrane. One of us previously documented transporter-mediated absorption from the apical membrane of the intestine of the bat *Artibeus jamaicensis* [17]. Paracellular absorption involves movement of solutes through a restrictive aqueous channel in the tight junctions of adjoining cells [18] by diffusion or by the process of solvent drag [19]. There are no published studies on this feature in microchiropteran bats, and so the goal of the present study was to measure the passive absorption that occurs in such a bat species. Our hypothesis was that the capacity for paracellular nutrient absorption would be much higher than previously measured in other nonflying mammals, as a compensatory mechanism for relatively lower intestinal nominal surface area, and perhaps would be as high as observed in bird species studied so far [12]–[16]. Although a plausible alternative hypothesis is for higher transporter site density per unit nominal area, transporter proteins may already be crowded into the intestinal cell membrane [20], and the mediated glucose transport activity over the entire small intestine of *A. jamaicensis* was unremarkable - the modal value among 13 mammalian species measured under similar conditions [17] and corrected for body mass differences. If our hypothesis were supported, studies on more species of bats will be necessary to test rigorously the idea that bats, as a group, differ from nonflying mammals in regards to paracellular absorption. Besides its contribution to nutrient absorption, passive, paracellular absorption has physiological and ecological importance as a pathway for absorption of appropriately-sized toxins made by humans (e.g., carbamate insecticides, glyphosate herbicide) and naturally occurring toxins in foods (e.g., caffeine, nicotine, some flavonoids).

We used a standard method in pharmacokinetics [21] to measure absorption in intact *Artibeus lituratus*, the great fruit-eating bats. In these experiments, compounds are assayed in serial blood samples in the minutes following either feeding or injection of a solution containing the compounds. To measure passive, paracellular absorption, we used water soluble compounds that are metabolically inert and lack affinity for intestinal mediated uptake mechanisms. Our test solutions included inert carbohydrates of two sizes (L-rhamnose, 164 Da; cellobiose, 342 Da), because paracellular absorption declines with increasing molecular size of the transported solute due to the pathway's sieve-like qualities [16]. For comparison of the paracellular, passive absorption with transcellular, transporter-mediated active absorption, we also included in test solutions another compound, explained as follows.

It is possible in experiments such as these that compounds not actively transported might be absorbed at a much slower rate than actively transported D-glucose, but over the entire length of the intestine and the extended time of digesta residence in the gut their absorption could still be fairly complete, implying a similar absorption rate to that of D-glucose [22]. An elegant approach to resolving this issue is to compare simultaneously in intact animals the extent and time course of absorption of compounds absorbed only passively vs. D-glucose or its analogue absorbed actively and passively. For example, in laboratory rats, the absorption rate of the nonmetabolizable, actively transported 3OMD-glucose apparently exceeded that of L-glucose, which is absorbed only passively, by about 9∶1, implying that most glucose was absorbed actively [9]. Similar conclusions have been drawn for dogs [10] and humans [11], but in house sparrows we previously found a ratio close to 1∶1, implying that the majority of glucose absorption was passive [23]. In order to apply this technique to the great fruit-eating bats, we included in our test solutions the nonmetabolizable analogue of D-glucose, 3-O-methyl-D-glucose (3OMD-glucose; MW = 194 Da), which has affinity for the D-glucose transporter(s) in the intestinal membrane.

Thus, based on earlier findings and the features of paracellular absorption, we tested three predictions for great fruit-eating bats as regards intestinal absorption. First, we predicted that the extent of absorption (i.e., fractional absorption or bioavailability) of the nonactively transported compounds would be inversely related to their molecular weights. Second, we predicted that the fractional absorption of these compounds would be much higher than previously measured in other mammals, and perhaps be as high as observed in birds. Finally, if the majority of glucose is absorbed passively in great fruit-eating bats, then absorption of 3OMD-glucose will be similar in rate and extent to that of the nonactively transported compounds, after adjustment for differences in molecular size.

## Results

### Paracellular absorption

**Figure 1:**
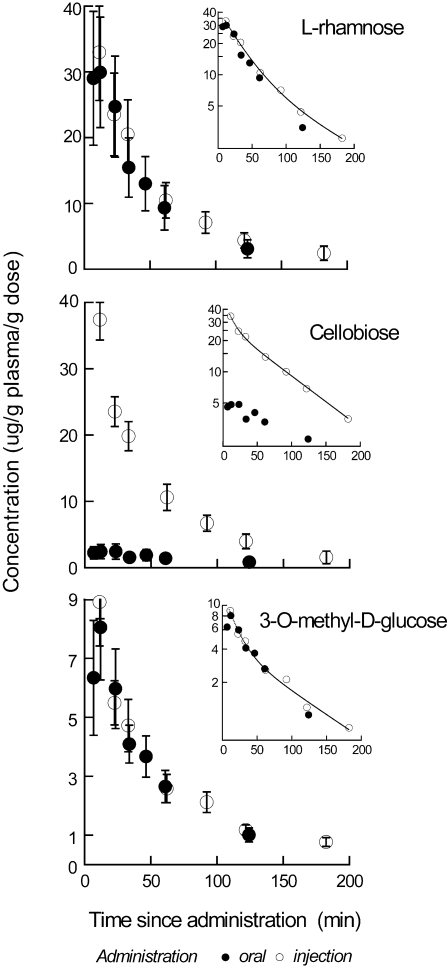
Pharmacokinetics of carbohydrates in plasma after oral administration and injection. Plots of mean (±s.e.m.) plasma L-rhamnose (upper plot), cellobiose (middle) and 3-O-methyl-D-glucose (lower plot) concentration as a function of time since each compound was consumed (oral) or injected into bats (*n* = 8). Each concentration (µg g^−1^ plasma) was normalized to the dose administered to the bat. Each inset displays the mean values on a semi-log plot. The line through points from the injection trial is the nonlinear fit to the biexponential model: *C_t_* = A e^−αt^+B e^−βt^ (see Methods, and Table 1 for derived parameters).

When the bats were fed solution with L-rhamnose, cellobiose, or 3OMD-glucose, the average concentration of each of the carbohydrates in the plasma peaked at around 10 min and then declined exponentially (Fig. 1). Evaluation of the decline (e.g., whether mono- or biexponential) is best accomplished using post-injection plasma data which are not influenced by the early rise in plasma concentration due to absorption kinetics. When solution was injected, plasma values declined exponentially beginning at the first sampling time, 10 min. Semi-log plots of injection data (insets of Fig. 1) had significantly better fits to a model of biexponential than monoexponential decline for L-rhamnose (F_2,3_ = 15.8, P<0.05), cellobiose (F_2,3_ = 15.0, P<0.05), and 3OMD-glucose (F_2,3_ = 12.1, P<0.05). The parameters from the biexponential fits (Table 1) were used subsequently to calculate the time course of absorption (below).

**Table 1:** Summary statistics on parameters for bi-exponential fit of plasma concentrations from injection/elimination experiments shown in Figure 1.

Parameter	L-rhamnose	Cellobiose	3OMD-glucose
A, µg g^−1^ plasma g^−1^ dose	32.9±6.0	37.9±14.0	9.25±4.47
B, µg mg^−1^ plasma g^−1^ dose	10.1±8.5	29.2±2.4	4.42±1.79
α, min^−1^	0.031±.011	0.0958±0.033	0.060±0.040
β, min^−1^	0.0081±0.004	0.0160±0.0006	0.0098±0.0028

The model was *C_t_* = A e^−αt^+B e^−βt^.

Mean concentrations for each probe (based on measurements in 8 bats) were fit to the model.

It is apparent from visual comparison of areas under the curve (AUC) for the oral vs. injection administration (Fig. 1) that fractional absorption was least for cellobiose. Although there is merit in this visual inspection of the patterns in these plots, because it affords the reader a simple and direct way to evaluate the data, more quantitative and sometimes less intuitive patterns emerge from calculations of fractional absorption (the quotient of AUC_oral_/AUC_inj_). Fractional absorption (*f*) varied significantly among the compounds (repeated measures ANOVA F_2,14_ = 49, P<0.001). The relatively low absorption of cellobiose was borne out (*f* = 0.10±0.03, *n* = 8), and it was significantly lower than for either 3OMD-glucose (0.96±0.11) or L-rhamnose (0.90±0.11) (post-hoc Tukey, P<0.001 in both cases). The absorption of 3OMD-glucose and L-rhamnose did not differ from each other (P>0.8).

**Figure 2:**
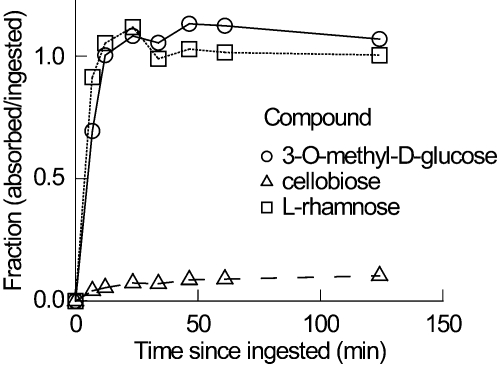
Cumulative intestinal absorption of carbohydrates after oral administration. The cumulative absorption as a function of time since ingestion of 3-O-methyl-D-glucose (3OMD-glucose; unfilled circles, solid line), cellobiose (unfilled triangle, dashed line) and L-rhamnose (unfilled square, dotted line).

The time course over which absorption of the carbohydrates occurred was derived by the method of Loo-Riegelmann [24], using kinetic constants derived from the injection plots (Table 1) and the mean plasma concentrations following oral administration of each compound (Fig. 1). Absorption of both 3OMD-glucose and L-rhamnose appeared to occur over the same time course and was completed by 12 min post-ingestion, according to the Loo-Riegelman analysis, whereas cellobiose absorption was nearly completed at 61 min post-ingestion (Fig. 2). To estimate how much absorption of 3OMD-glucose was passive, we assumed that absorption of L-rhamnose is a proxy for passive absorption of 3OMD-glucose, once adjusted for the small difference in MW. Because diffusion in water declines with MW^1/2^ [25], each value of L-rhamnose absorption was decreased by 8% ( = 100·[194^1/2^−164^1/2^]/194^1/2^). Assuming that the absorption of 3OMD-glucose represents the sum of passive+mediated absorption, the ratio of the amounts absorbed (L-rhamnose/3OMD-glucose) indicates the proportion of 3OMD-glucose absorption that occurs via the passive pathway. Considering the similar time course of absorption of the two compounds, and the ratio of *f*_L-rhamnose_/*f*_3OMD-glucose_ (0.88±0.07, *n* = 8; corrected for difference in MW), the vast majority of 3OMD-glucose absorption was apparently passive.

### Intestinal morphometrics

**Figure 3:**
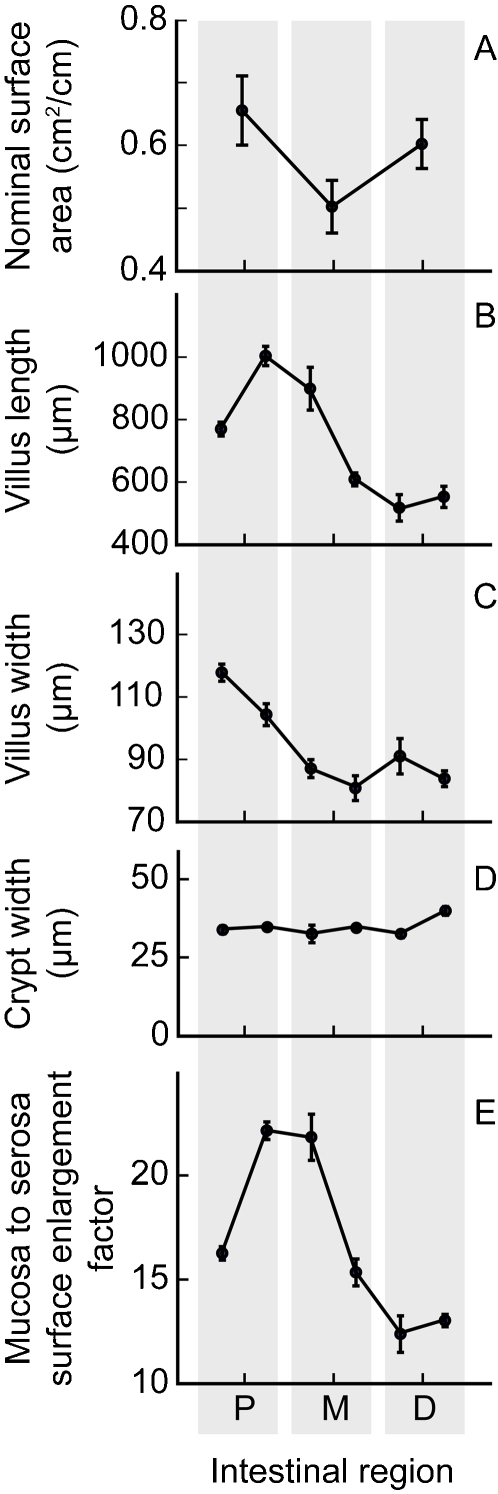
Morphometrics of the small intestine of great fruit-eating bats. Nominal surface area of the entire small intestine (*n* = 3), based on measures in the proximal (P), mid (M), and distal (D) regions (Fig. 3A) was 28.13±2.89 cm^2^. The mucosa (i.e., villous) to serosa (i.e., nominal) surface enlargement factor, which varied significantly with intestinal position (Fig. 3E), was calculated from measures of villus length (Fig. 3B; also varied significantly with position), villus width (Fig. 3C), and crypt width (Fig. 3D). Villi, overall, increased intestinal surface area by 16.8±1.0 times.

Intestinal morphometrics were studied in three great fruit-eating bats (mean mass 69.6±5.7 g). Mean stomach wet mass was 0.40±0.01 g. At a gross scale, there was no obvious division into small and large intestine, as also reported in six other morphometrics studies in bats (reviewed by [26]). Mean whole intestinal mass and length were 2.46±0.23 g and 47.8±2.0 cm, respectively. Nominal surface area for the entire intestine was 28.13±2.89 cm^2^ (Fig. 3). Dimensions for villus length and width, and for crypt width, were used to calculate the mucosal to serosal surface enlargement factor, which varied significantly with intestinal position (Fig. 3). Overall, the villi increased intestinal surface area by 16.8±1.0 times.

## Discussion

### Paracellular absorption in great fruit-eating bats compared with nonflying mammals

As expected, absorption by intact bats of metabolically inert, nonactively transported water soluble probes L-rhamnose and cellobiose declined with the increasing MW of the probes. This pattern is apparent in other mammals, but fractional absorption is significantly higher in great fruit-eating bats (Table 2). We used cellobiose for a molecule relatively large in molecular radius whereas many other studies used lactulose. We do not think that this difference confounds our comparison because the two compounds are identical in MW, but cellobiose is composed of two molecules of glucose whereas lactulose has one molecule each of galactose and fructose. But, even if the comparison is restricted only to L-rhamnose, which was measured in all the species, the fractional absorption measured for that compound in great fruit-eating bats, 0.9, is well above the upper bound of the 95% confidence interval for L-rhamnose fractional absorption in the nonflying mammals (0.69 for experiments with nutrient present in the lumen, 0.21 for experiments without).

**Table 2:** Comparison of fractional absorption of nonactively transported, water soluble compounds by intact small mammals.

Species	Nutrients Present in oral solution?	Fractional absorption (µg absorbed/µg consumed)		Reference
		L-rhamnose	cellobiose or lactulose	
		MW = 164	MW = 344	
Bats				
Great fruit-eating bat (*Artibeus lituratus*)	Yes	0.90±0.11	0.1±0.03^1^	This study
Egyptian fruit bat (*Rousettus aegyptiacus*)	Yes	0.62±0.04	0.22±0.04^1^	[29]
Non-flying mammals				
Laboratory rat (*Rattus norvegicus*)	Yes	0.22±0.05	0.09±0.014	[30]
Laboratory rat (*Rattus norvegicus*)	No	0.25±0.05	0.05±0.0092	[30]
Laboratory rat (*Rattus norvegicus*)	No	0.028	0.0044	[63]
Laboratory rat (*Rattus norvegicus*)	No		0.0207±0.006	[64]
Laboratory rat (*Rattus norvegicus*)	No		0.012±0.005	[65]
Laboratory rat (*Rattus norvegicus*)	No		0.0102	[66]
Guinea pig (*Cavia porcellus*)	No		0.03±0.011	[64]
Guinea pig (*Cavia porcellus*)	No	0.064	0.023	[63]
Hamster (*Mesocricetus auratus*)	No	0.0235	0.0055	[63]
Cuis (*Galea musteloides*)	Yes	0.163±0.046	0.079±0.0145^1^	[67] and E. Caviedes-Vidal, unpubl. data

1 compound was cellobiose whereas other studies used lactulose.

Passive absorption in great fruit-eating bats is as high as measured previously in various avian species range 0.65–0.9) using metabolically inert, nonactively transported water soluble probes such as L-rhamnose [16], L-glucose [12], [13], [15], and mannitol [27], [28]. Relatively extensive paracellular absorption might be a feature in bats generally. In another study on Egyptian fruit bats (*Rousettus aegyptiacus*) from the other major clade of bats (Megachiroptera), we also found relatively high fractional absorption of nonactively transported water soluble compounds [29] (Table 2). Small flying bats and birds may share the characteristics of relatively small guts with less nominal surface area and relatively high paracellular absorption, compared with similar sized nonflying mammals [30].

Paracellular absorption is enhanced when active sugar or amino acid transport occurs concurrently, i.e., when these nutrients are in the lumen, and this pattern is apparent in the data in Table 2. Although this enhancing effect probably occurred in our experiment because our solutions contained 50 mM 3-O-methyl-D-glucose, the very much higher fractional absorption we measured for L-rhamnose by great fruit-eating bats, compared with results in nonflying mammals, cannot be easily explained as due to this effect, because it increases fractional absorption by a relatively smaller amount, about 10 percentage points (6±3%, *n* = 4 studies with L-rhamnose, L-glucose, and mannitol [16], [28], [30]. The mechanism(s) for acceleration by luminal nutrients might be increased solvent drag and/or cytoskeletal contractions [31]–[34] or protein strand alterations that alter the tight junction effective pore size [19].

The difference in fractional absorption between the bat and the nonflying mammals is much reduced for the large MW probes. This might reflect that organisms in both groups have tight junctions with similar effective pore size that becomes restrictive for molecules not much larger than the radius and MW of cellobiose and lactulose.

### Paracellular absorption can account for the majority of glucose absorption

Great fruit-eating bats also differ from the nonflying mammals in that paracellular absorption apparently accounts for the majority of glucose absorption. In simultaneous measurements of absorption of actively transported D-glucose or 3OMD-glucose and passively transported L-glucose [35] in rats [9], dogs [10], [36], and humans [11], D-glucose or its analog 3OMD-glucose were absorbed about ten times faster than L-glucose, implying that more than 90% of glucose absorption was mediated. Our simultaneous measurements using L-rhamnose as a proxy for nonmediated absorption of 3OMD-glucose imply that nearly 90% of 3OMD-glucose absorption is passive in great fruit-eating bats. Schwartz et al. [22] made the point that simply comparing fractional absorption for actively transportable vs. nonactively transportable compounds might be misleading. Suppose that 3OMD-glucose is absorbed at a high rate in the proximal portion of the intestine, whereas L-rhamnose is absorbed at a very slow rate. The fractional absorption of L-rhamnose could still be fairly complete if its slow absorption occurs over the entire length of the intestine and over the entire time of digesta residence. We do not think that this explanation applies to great fruit-eating bats. L-rhamnose absorption normalized to MW^1/2^ (to account for MW dependent differences in diffusion) did not seem slow or prolonged compared with that for 3OMD-glucose (Fig. 2). 3OMD- and L-rhamnose had apparent absorption rates similar to each other throughout all the sampling time points (Fig. 2). Hence, we conclude that the vast majority of 3OMD-glucose was absorbed passively via the paracellular pathway.

### Mechanism for high paracellular absorption in great fruit-eating bats

At least three mechanisms might account for higher paracellular absorption in great fruit-eating bats, or bats generally. First, increased villous area per unit intestinal nominal surface area might be associated with more cell junctions across which paracellular transport occurs, if villus lengths are increased mainly by increase in number of similar-sized enterocytes. Second, increase in effective pore radius in the junction, caused by differences in claudins and other proteins that create the sieving effect, will increase paracellular permeation over certain size ranges of molecules [16], [37]. Third, increase in water flux across the tight junction will increase solute permeation by increased solvent drag [19], [38]. We have no information on solvent drag, and the similar pattern of decline in fractional absorption with increasing probe MW in great fruit-eating bats and nonflying mammals (Table 2) suggests no major difference in effective pore size. There may be evidence for increased villous area per unit intestinal nominal surface area in bats.

The ratio of villous area relative to nominal area, sometimes called the surface enlargement factor (SEF), has been previously measured in some bat species [39]–[41] and in nonflying mammal species [39], [42]–[50] by a number of investigators using a variety of methods. Including our data on great fruit-eating bats, SEF does not change significantly with increasing body mass (both log_10_ transformed; F_1,22_ = 4.09, P>0.05), but is significantly higher in bats (SEF = 9.5±1.9, *n* = 6 species) than in nonflying mammals (4.8±0.5, *n* = 19 species) (F_1,23_ = 12.6, P<0.002). We caution that this measurement is sensitive to the particular method used by an investigator [51]. For example, the coefficient of variation ( = S.D./mean) for multiple measurements by different investigators in mice, rats, dogs, and cats is relatively high (0.48±0.02). However, the measure of SEF on great fruit-eating bats (16.8) is about double that made by one of us using the same methodology in Sprague Dawley rats (7.5±0.7, *n* = 4; S.R. Lavin and W.H. Karasov, unpubl. data). Also, two other investigators (R.E. Barry, Jr. and T.M. Mayhew) have made measurements on a bat species and a similar-sized nonflying mammal species using uniform methodology [39], [41], [48], and in both cases the SEF in the bat species exceeded that in the nonflying mammal by ≥59%. Thus, we tentatively conclude that bats, relative to nonflying mammals, exhibit an enhancement of the villi, which could partly account for relatively high paracellular absorption in great fruit-eating bats.

### Far-reaching consequences of differences in intestinal absorption

From an evolutionary perspective, one can argue that there are both costs and benefits to intestinal permeability to hydrosoluble biochemicals, which would explain why it is exhibited by some but not all mammals. A possible cost is that a high intestinal permeability that permits passive absorption might be less selective than a carrier-mediated system for nutrient absorption and might permit toxins to be absorbed from plant and animal material in the intestinal lumen [20]. We have suggested that a selective advantage in the case of bats is that it can compensate for shorter intestine and lower nominal surface area, and Pappenheimer [52] suggested that passive absorption may confer a selective advantage because it requires little energy and provides a mechanism whereby rate of absorption is matched to rate of hydrolysis. Opposing costs and benefits can lead to variation among species in intestinal permeability to hydrosoluble biochemicals. For small bats with high intestinal permeability, vulnerability to hydrophilic toxins could be an important ecological driving force, constraining food exploratory behavior, limiting the breadth of the dietary niche, and selecting for compensatory behaviors such as searching for and ingesting specific substances that inhibit hydrophilic toxin absorption [53].

## Materials and Methods

### Bats, and their maintenance

Experiments were conducted on 5 adult male and 3 adult female great fruit-eating bats (mean masses±SEM, respectively, 81.9±3.8 and 79.3±3.5 g) held in large outdoor flight cages at the campus of the Universidade Estadual Paulista in Rio Claro (Brasil). Bats were provided with fruits (bananas, apples and papayas) plus vitamin supplement (Aminomix pet, Vetnil Indústria e Comércio de Produtos Veterinários Ltda, Brasil) and water ad libitum. All animal procedures adhered to institutional animal use regulations and approved animal use protocols.

### Measurement of absorption

Fractional absorption, or bioavailability, of test carbohydrates (“probes”) was calculated using standard pharmacokinetic procedures [see below; [16]]. Probes were administered to animals in two separate trials by: 1) voluntary feeding from a syringe (oral), and 2) intraperitoneal injection. Order of treatment was randomly assigned and a minimum of one week separated trials on individual animals. Oral doses of the following probes were administered in an isosmotic solution (640.7±16.8 µl or approximately 0.8% of body mass) that also contained 25 mM NaCl, 3-O-methyl-D-glucose (86 mg kg^−1^ body mass), L-rhamnose (81 mg kg^−1^ body mass), and cellobiose (254 mg kg^−1^ body mass). Injected doses were delivered in a sterile isosmotic solution (269±8 µl or approximately 0.33% of body mass) as follows: 3-O-methyl-D-glucose (48 mg kg^−1^ body mass), L-rhamnose (45 mg kg^−1^ body mass), and cellobiose (127 mg kg^−1^ body mass). A series of small blood samples (≤80 µl) were taken from the superficial veins from wings and legs at 6.9±0.3 min, 12.1±0.5 min, 23.4±0.8 min, 33.8±0.7 min, 46.5±0.5 min, 61.0±0.9 min, and 124.2±2.0 min post-administration. A background blood sample was also taken before administration of probes.

### Sample analysis

Blood plasma was separated from cells using standard heparinized hemo-capillary tubes (Fisher Scientific, Pittsburg, PA, USA) and a micro-hematocrit centrifuge. Plasma mass was determined to 0.1 mg and samples were deproteinated using acetonitrile (with a ratio of plasma: H_2_O: acetonitrile of 1∶7∶24 in volume; modified from [54]. Briefly, 210 µl of distilled H_2_O+720 µl of acetonitrile (HPLC grade) were added to 30 µl of plasma. The mixture was vortexed for 20 s and then centrifuged at 10,000 *g* for 2 min. The pellet was discarded, the supernatant dried at 50°C and stored frozen until analysis.

Carbohydrate probes were derivatized for high performance liquid chromatography (HPLC) fluorescence detection by reductive amination with anthranilic acid (2-aminobenzoic acid), following [55], [56] with minor modifications. Briefly, previously dried plasma samples were reconstituted with 50 µl 1% sodium acetate solution and mixed with 50 µl of anthranilic acid reagent solution. The anthranilic acid reagent consisted of 30 mg ml^−1^ anthranilic acid and 20 mg ml^−1^ sodium cyanoborohydride dissolved in a previously prepared solution of 4% sodium acetate^.^3H_2_O and 2% boric acid in methanol. Samples were transferred to screw-cap glass autosampler vials which were tightly closed and heated at 65°C for 3 hours. After cooling to ambient temperature, 500 µl of HPLC solvent A was added to vials, which were mixed vigorously in order to expel the hydrogen gas evolved during the derivatization reaction.

Carbohydrate derivatives were separated on a Waters Pico·Tag® C-18 reversed phase HPLC column (3.9×150 mm, 5 µm, Waters Corporation, Milford, MA, USA) using a 1-butylamine-phosphoric acid-tetrahydrofuran mobile phase system. The separations were carried out at room temperature using a flow rate of 1 ml min^−1^. Solvent A consisted of 0.2% 1-butylamine, 0.5% phosphoric acid, and 1% tetrahydrofuran (inhibited) in HPLC grade water (18.3 MΩ resistance, Barnstead EasyPure UF System) and solvent B consisted of equal parts solvent A and HPLC grade acetonitrile. The elution gradient was 90% to 82% of solvent A in 18 minutes. The column was washed during 10 minutes with 100% solvent B and equilibrate for 10 minutes at initials conditions. The HPLC system consisted of a Beckman automated binary system with a pump (model 126), an autosampler (model 507) and an interface (Model 406). Derivatives of carbohydrate probes in samples and standard solutions were detected with a fluorescence Detector Gilson Model 121 (Gilson, Inc) with a excitation filter of 305–395 nm and emission filter of 450 nm (bandpass 40 nm). Limits of detection for all probes in water were 1–2 ng loaded onto the HPLC column. Anthranilic acid and sodium cyanoborohydride were obtained from Sigma-Aldrich (St. Louis, MO, USA). Acetonitrile and tetrahydrofuran HPLC grade were obtained from JTBaker (Mallinckrodt Baker, Inc. Phillipsburg, NJ, USA), 1-butylamine and phosphoric acid from Anedra (Anedra S.A. Buenos Aires, Argentina).

### Pharmacokinetic calculations

For each compound, the concentration in each plasma sample at time *t* was normalized to the weight of each sample (*C_t_*, µg g^−1^ plasma) and plotted against sampling time since the compound was administered either orally or by injection. The integration of the area under this curve (*AUC_t_*) represents the amount of compound that has been absorbed from time 0 up to time *t*, whereas *AUC_total_* denotes the total amount of compound absorbed from 0 up to infinity time (∞). Following typical procedures in pharmacokinetics [21], the area from *t* = 0 to *t* = *x* min (when the final blood sample was taken) was calculated using the trapezoidal rule. The area from *t* = *x* min to *t* = ∞ was calculated as *AUC* ^x→∞^ = *C_t_* (at *t = x*)*/k*, where *k* is a rate constant which can be determined for each bat in each experiment based on the terminal portion of its absorption curve. The total *AUC* ^0→∞^ was obtained by summing the two areas. Fractional absorption (*f*), or bioavailability, for each compound was estimated based on the ratio between the area under the plasma concentration versus time curve for oral administration experiments (*AUC_oral_*, in units of ng·min·g plasma^−1^) and injection experiments (*AUC_inj_*) normalized to respective dosage given to the animal:
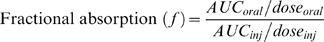
(1)This method of calculating *f* is favored because it makes no major assumptions about compartments or kinetics. Fractional absorption estimates how much of the ingested probe was absorbed into the animal's system. The calculations of *f* and their statistical comparison (below) were performed based on data for individuals, although data shown in figures are mean values corrected for differences in dose between individuals.

Additional analyses relating to the time course of absorption were made assuming an open two-compartment model and first order elimination. As described in Results, a bi-exponential elimination model fit the data on mean *C_t_* vs. sampling time post injection significantly better (*P*<0.05) than a mono-exponential model, using an F-test [57]. For each compound, parameters for the model were derived by nonlinear regression, fitting the data to the biexponential model:
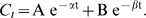
(2)In the two-compartment model, rate constants and distribution spaces are derived from the constants A, B, α, and β. For example, the elimination rate constant of the probe from the apparent central compartment is estimated as (A+B)/([A/α]+[B/β]). The rate constants and spaces were used in conjunction with the data from ingestion experiments to calculate the cumulative proportion of *f* that was absorbed at each blood sampling time point (*P_t_*), according to the Loo-Riegelman method [24], [21]. The true cumulative absorption at any sampling time point (*t*) is the product of *f* and *P_t_*. Wagner [58] considered the two-compartment open model to be the “minimum” model for linear systems in pharmacokinetics, but noted that if the elimination kinetics tended towards a monoexponential decline, hence indicating a one-compartment model, then the Loo-Riegelman method “collapses” into the Wagner-Nelson method for calculating absorption for a one-compartment open model, giving similar results. The physiological argument for the two-compartment approach is that if there are some organs and tissues in an organism where blood circulation is not as high as the rest of body, this may lead to a higher concentration of probe in these peripheral compartment(s) compared to the central compartment. Thus these poorly perfused peripheral compartments can serve as another source for probes entering the central compartment, secondary to an absorption site like the small intestine. In comparison to the one-compartment model, a two-compartment model is more conservative in that it provides corrections for the redistribution of probe from these peripheral compartments [21], [59].

### Analysis of intestinal morphometrics

Three bats (mean mass 69.58±4.69 g) were anesthesiated with ketamine (40 mg/kg body weight) and acepromazine (0.5mg /Kg body weight) and after removing the gastrointestinal tract and, still under deep anesthesia effect, were killed by decapitation. The stomach and small intestine were dissected out, the lumen washed with ice cold 1% NaCl solution to remove digesta, and then the intestine was blotted dry and weighed and measured for length. Immediately, the intestine was divided in three equally portions, the proximal, medial and distal regions. The pieces were carefully cut longitudinally with small surgical blunt scissor, opened, cleaned with ice cold 1% NaCl solution and placed with the mucosa side up on a cold stainless steel platine. Using a digital caliper, two measures of length and four of width were taken to estimate nominal surface area of each portion. For histological examination, six one-centimeter pieces, two from each region were cut and immersed in 10% formalin solution for two hours. Before embedding, tissue samples were dehydrated through a graded series of ethanol solutions and embedded in hydroxyl methacrylate. We sectioned thirty 2-µm thick sections of each tissue sample. Sections were mounted on slides, stained with hematoxylin-eosine and covered with cover glasses. Microphotographs were taken using an Olympus BX50 microscope connected to a video-camera (Sony CCD-Iris) and a PC-based image analysis system (ImageJ, [60]). From each section we measured the circumference of the serosal surface, length and width of villi and the width of the cripts. We took 30 such measurements per section, resulting in 90 measurements per individual. We measured only those villi that were cut in their midline, from tip to base, as verified by observations of similarly sized and shaped enterocytes. These data were used to estimate the surface area enlargement factor (SEF) [61]. To avoid inflation of degrees of freedom by repeated measurements within individuals, means and standard deviation were calculated for individual bats. These means were used in statistical analyses.

### Statistical analysis

Numerical data are presented as means±1 SEM (*n* = number of animals). Fractional absorption (*f*) of the three compounds, measured within each bat, was analyzed by repeated measures analysis of variance (ANOVA; [62]), followed by Tukey multiple comparisons test. The F-values of these and other ANOVAs are presented in the text with the relevant degrees of freedom as subscripts. Variation in morphometric measures along the intestine was evaluated with non parametric U-Mann-Whitney test with correction for small sample size. Nonlinear curve fitting (Gauss-Newton algorithm, SYSTAT; [62]) was used to fit kinetic data, and kinetic models were compared according to [57]. Riviere (1999) points out that regression analysis of pharmacokinetic data based on minimization of residual sums of squares (SS) are completely influenced by the earlier and greater concentrations, which leads to overestimation of most of the measurements made at later time points. We checked for this by inspecting the pattern of residuals, and corrected for it when necessary by weighting the regression analysis by 1/*C_t_* ^2^, as recommended [59]. This procedure, which ameliorates the problem, slightly changes the derived kinetic parameters but did not affect our overall conclusions. Statistical significance was accepted for α<0.05.
